# QSAR-Guided Virtual Screening and Molecular Dynamics Reveal Olaparib as a Repurposing Lead Against α-Synuclein Aggregation

**DOI:** 10.3390/ijms27157025

**Published:** 2026-08-05

**Authors:** Mena Abdelsayed, Yassir Boulaamane

**Affiliations:** 1 Lankenau Institute for Medical Research, Philadelphia, PA 19096, USA; 2 Laboratory of Innovative Technologies, National School of Applied Sciences of Tangier, Abdelmalek Essaadi University, Tetouan 93000, Morocco

**Keywords:** α-synuclein, Parkinson’s disease, drug repurposing, QSAR, ChemBERTa, molecular docking, AutoDock Vina, applicability domain, molecular dynamics, MM-PBSA

## Abstract

Parkinson’s disease (PD) is characterised by the pathological aggregation of α-synuclein (α-syn) into Lewy body inclusions, yet no disease-modifying therapy exists. To address this, we developed an integrated computational pipeline combining quantitative structure–activity relationship (QSAR) modelling, structure-based virtual screening, molecular dynamics (MD) simulation, and molecular mechanics Poisson–Boltzmann surface area (MM-PBSA) binding free energy calculations to repurpose FDA-approved drugs as α-syn fibril inhibitors. Two complementary QSAR model families were trained on 501 α-syn binding affinity records from BindingDB: Morgan extended-connectivity fingerprint (ECFP4) classifiers and a frozen ChemBERTa-77M-MLM transformer encoder, each using Random Forest and Logistic Regression. The applicability domain (AD) was assessed using Morgan–Tanimoto similarity (Tc ≥ 0.40) and calibrated ChemBERTa cosine distance (θ ≤ 0.367). A three-stage funnel applying central nervous system (CNS) permeability filters, a consensus QSAR probability threshold (≥0.80), and AD gating reduced 2241 FDA-approved drugs to 205 candidates for AutoDock Vina 1.2.6 docking against two sites on the cryo-electron microscopy (cryo-EM) α-syn fibril structure, PDB 6SSX: the inter-protofilament cleft (Site 1) and the non-amyloid-beta component (NAC) groove (Site 2). The Morgan fingerprint models achieved an area under the receiver operating characteristic curve (AUROC) of up to 0.940 and a balanced accuracy of 0.810; the ChemBERTa models achieved an AUROC of 0.785 and a balanced accuracy of 0.728. Notably, ChemBERTa AD covered 76.8% of the FDA drugs versus only 5.5% for Morgan–Tanimoto, enabling broad-spectrum screening. The top docking candidates were Olaparib (−7.91 kcal/mol), Paliperidone (−7.75 kcal/mol), Niraparib (−7.18 kcal/mol), Dordaviprone (−7.06 kcal/mol), and Parecoxib (−6.89 kcal/mol). The MD simulations over 200 ns across three independent replicates confirmed stable NAC groove binding, and replicate-averaged MM-PBSA calculations yielded ΔG = −20.6 ± 1.9 kcal/mol for Olaparib at Site 2, −17.1 ± 0.9 kcal/mol for Risperidone, and −16.9 ± 0.8 kcal/mol for Paliperidone, reported as the mean ± standard error of the mean (SEM) across replicates. Olaparib additionally formed five hydrogen bonds in the representative pose, while MD trajectories maintained approximately 2–5 hydrogen bonds, together with a halogen bond within the NAC groove, the largest contact count of any screened compound. These findings identify Olaparib as a novel high-affinity repurposing lead, while Paliperidone and Risperidone are reported as chemically informative secondary NAC–groove binders rather than proposed antiparkinsonian therapeutics, given that their dopamine D2-antagonist pharmacology is clinically associated with drug-induced parkinsonism. All of the candidates warrant experimental validation via thioflavin-T fluorescence or nuclear magnetic resonance (NMR) spectroscopy.

## 1. Introduction

Parkinson’s disease (PD) affects more than six million individuals worldwide and is projected to double in prevalence by 2040 as populations age [1]. It is characterised clinically by motor symptoms, including bradykinesia, rigidity, and resting tremors, as well as non-motor manifestations, including cognitive decline and autonomic dysfunction [2,3]. The pathological hallmark of PD is the presence of Lewy bodies and Lewy neurites whose primary protein component is α-synuclein (α-syn), encoded by the *SNCA* gene [4,5,6]. Point mutations in *SNCA* (A53T, A30P, and E46K) and gene duplication/triplication cause familial PD, and genome-wide association studies consistently implicate the *SNCA* locus in sporadic PD, underscoring the central role of α-syn in disease aetiology [7,8].

Under pathological conditions, monomeric α-syn (140 residues; natively unstructured) undergoes a conformational transition through oligomeric intermediates into amyloid fibrils [9]. The non-amyloid-beta component (NAC) region spanning residues 61–95 constitutes the aggregation nucleus and is essential for fibril formation, as deletion of residues 71–82 abolishes aggregation in vitro [6]. Recent cryo-electron microscopy (cryo-EM) studies have resolved multiple α-syn fibril polymorphs at near-atomic resolution, including PDB 6SSX (polymorph 2A, 3.0 Å), revealing distinct inter-protofilament interfaces and groove geometries that offer druggable binding pockets [10]. Disruption of fibril elongation at these sites represents a rational therapeutic strategy [11].

Despite decades of research, no disease-modifying therapy for PD exists [12]. De novo drug discovery is expensive, time-consuming, and associated with high clinical attrition. Drug repurposing identifies new therapeutic uses for approved drugs, circumventing many of these barriers by leveraging established safety profiles, pharmacokinetic data, and existing manufacturing infrastructure [13]. Computational approaches have accelerated repurposing by enabling rapid, large-scale screening prior to resource-intensive experimental assays. Quantitative structure–activity relationship (QSAR) modelling predicts biological activity from molecular structure, while structure-based virtual screening (molecular docking) estimates binding affinity of small molecules to defined protein sites [14]. Combining both approaches in a funnel architecture maximises the signal-to-noise ratio in candidate selection.

In this study, we present an integrated computational pipeline to identify FDA-approved drugs with potential α-syn aggregation inhibitory activity. Specifically, we: (i) curated a QSAR training set from BindingDB comprising 501 compounds with measured α-syn binding affinity; (ii) trained two complementary model families with calibrated applicability domain estimation; (iii) applied a three-stage virtual screening funnel to 2241 FDA-approved drugs; and (iv) performed AutoDock Vina docking of 205 filtered candidates against two structurally characterised binding sites on the 6SSX α-syn fibril. To our knowledge, this is the first α-synuclein fibril virtual-screening pipeline to combine a ChemBERTa transformer encoder with dual (Morgan–Tanimoto and ChemBERTa-embedding) applicability-domain gating, which together constitute the principal methodological novelty of this work relative to previous α-syn screening efforts.

## 2. Results

### 2.1. QSAR Model Performance

Querying BindingDB for α-synuclein (UniProt P37840) yielded 501 unique compounds after deduplication and salt stripping. Of these, 355 (70.9%) were classified as active (pKi ≥ 6.0) and 146 (29.1%) as inactive, representing a moderate class imbalance of 2.4:1. The Murcko scaffold–stratified split produced training (*n* = 316), validation (*n* = 81), and test (*n* = 104) sets with preserved activity ratios. Feature engineering reduced the raw descriptor matrix from 2258 to 849 features following variance and collinearity filtering.

**Figure 1:**
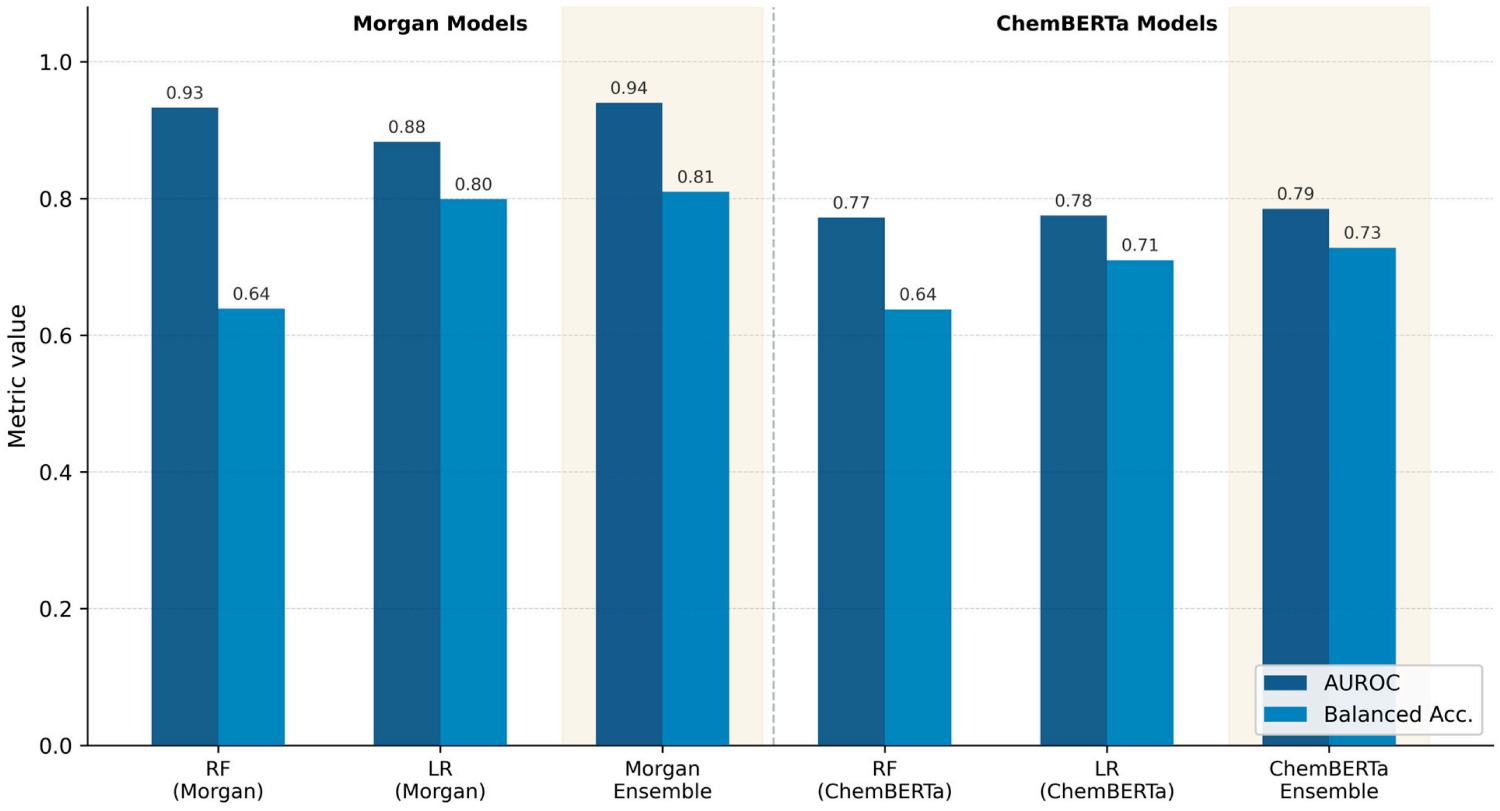
The QSAR model performance on the held-out test set (*n* = 104). The grouped bars show AUROC and balanced accuracy for each classifier. The Morgan-based models (**left**) and ChemBERTa-based models (**right**) are separated by a dashed line. The ensemble models are highlighted (gold background).

Among the Morgan-based models, XGBoost achieved the highest individual AUROC (0.94) and balanced accuracy (0.79), while the Morgan ensemble reached an AUROC of 0.94 and a balanced accuracy of 0.81. XGBoost was included as an exploratory gradient-boosted comparator and is, therefore, not shown in Figure 1, which reports the ensemble and individual classifier configurations (full metrics in Table S1). Logistic Regression showed the highest balanced accuracy among individual Morgan classifiers (0.80) at a lower AUROC (0.88). The ChemBERTa ensemble achieved an AUROC of 0.79 and a balanced accuracy of 0.73, reflecting the challenge of predicting α-syn activity from general-purpose transformer embeddings without task-specific fine-tuning. Figure 1 compares AUROC and balanced accuracy across all of the six model configurations (full metrics in Table S1).

### 2.2. Applicability Domain Analysis and Virtual Screening

**Figure 2:**
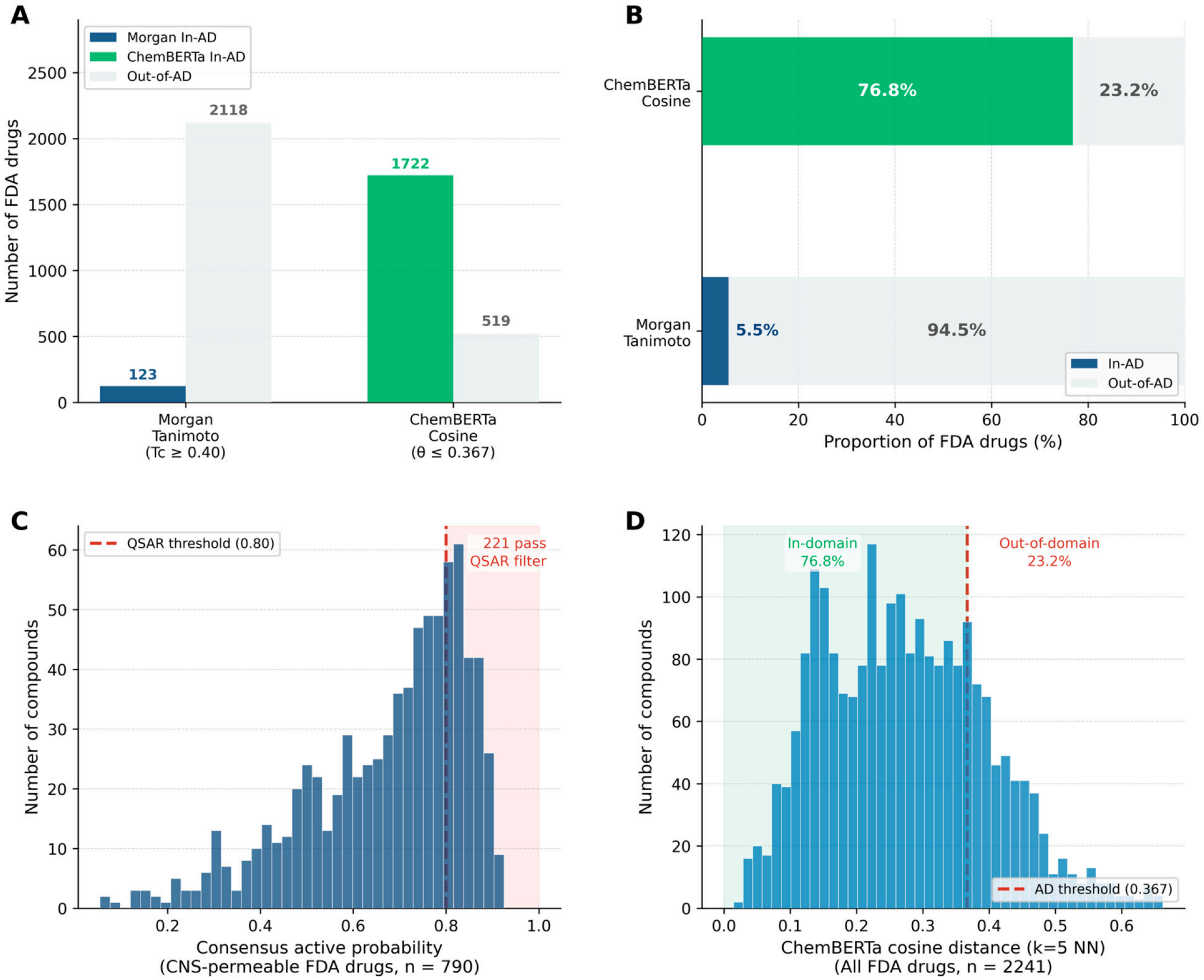
The applicability domain (AD) profiling and virtual-screening gating of the FDA-approved drug library. (**A**,**B**) A comparison of the absolute compound counts and relative percentages (%) within the AD, showing a 14-fold expansion in reliable chemical space via the ChemBERTa cosine distance (θ ≤ 0.367, 76.8%) relative to the Morgan–Tanimoto similarity (Tc ≥ 0.40, 5.5%). In (**B**), green denotes the ChemBERTa in-AD fraction, dark blue the Morgan–Tanimoto in-AD fraction, and light grey the out-of-AD fraction. (**C**) The distribution of consensus QSAR active probabilities across CNS-permeable drugs (*n* = 790), highlighting the 221 candidates passing the ≥ 0.80 threshold. (**D**) The distribution of ChemBERTa k = 5 nearest-neighbour embedding cosine distances across the full library (*n* = 2241) relative to the calibrated 0.367 domain boundary.

The Morgan–Tanimoto AD (Tc ≥ 0.40) covered only 123 of 2241 FDA-approved drugs (5.5%), reflecting the narrow chemical space of the BindingDB α-syn training set relative to the structural diversity of approved drugs. In contrast, the calibrated ChemBERTa cosine distance AD (threshold = 0.367) covered 1722 drugs (76.8%), a 14-fold expansion in coverage (Figure 2A,B). This dramatic difference arises from ChemBERTa’s pre-training on 77 million diverse PubChem SMILES, which generates embeddings that generalise far beyond the narrow structural neighbourhood of the training set.

Figure 2 summarises the screening funnel. The Morgan–Tanimoto similarity retains only a small fraction of the FDA library, whereas the ChemBERTa cosine-distance criterion keeps most compounds within a reliable prediction region. Among 790 CNS-permeable drugs, 221 exceeded the consensus QSAR cutoff, and ChemBERTa AD gating reduced this set to 205 compounds for docking. The final set, therefore, represents a small, high-confidence subset enriched for both CNS compatibility and predicted α-syn activity.

### 2.3. Molecular Docking Results

**Figure 3:**
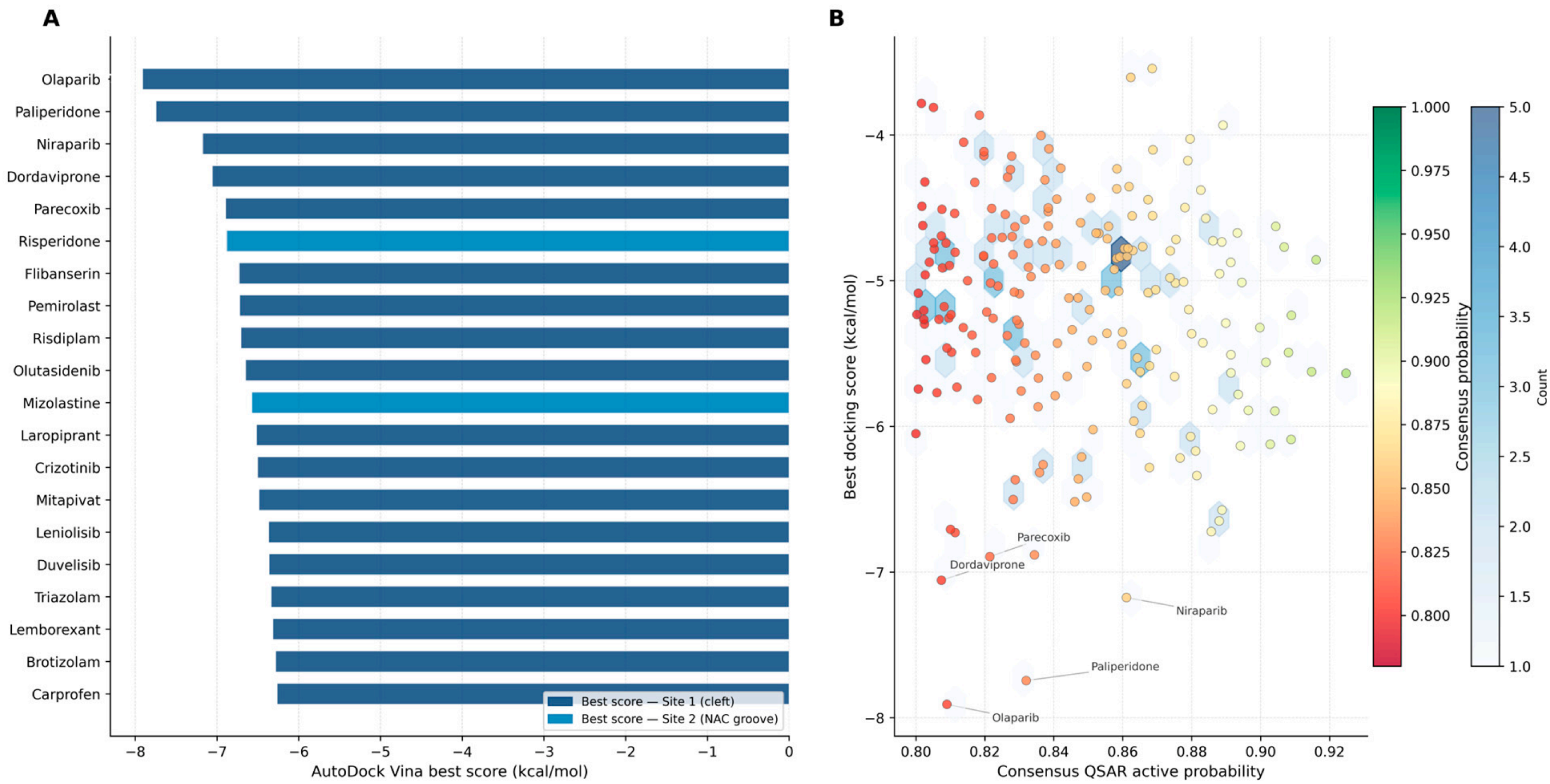
The structure-based molecular docking performance and QSAR-docking correlation for the 205 virtual screening candidates. (**A**) The top 20 FDA-approved drugs ranked by the best AutoDock Vina binding score, with dark blue bars denoting a preference for the inter-protofilament cleft (Site 1) and light blue bars indicating a preference for the NAC groove (Site 2). (**B**) A correlation plot mapping the best docking score against the consensus QSAR active probability across a density-binned hexbin grid (count scale, blue gradient), with individual points colour-coded by QSAR probability (red-to-green gradient) and the five top-ranked leads explicitly labelled.

All 205 candidates were docked against both binding sites of the 6SSX α-syn fibril. The inter-protofilament cleft (Site 1) was the preferred binding site for the majority of compounds (*n* = 178, 86.8%), whereas the NAC groove (Site 2) was preferred by 24 compounds (11.7%); the remaining three compounds (1.5%) did not yield a valid pose at either site. Figure 3A shows the top 20 ranked compounds by the best AutoDock Vina score, led by Olaparib (−7.91 kcal/mol), Paliperidone (−7.75 kcal/mol), Niraparib (−7.18 kcal/mol), Dordaviprone (−7.06 kcal/mol), and Parecoxib (−6.89 kcal/mol) (full top 20 in Table S2). Although Site 1 dominated overall across the screened library, several of the highest-ranked compounds, including Risperidone (−6.88 kcal/mol) and Mizolastine (−6.57 kcal/mol), achieved their best scores at Site 2, highlighting the NAC groove as a competitive pocket among top leads. Olaparib scored best at Site 1 (−7.91 kcal/mol) rather than Site 2 (−6.96 kcal/mol), so its Site 2 prioritisation was based on subsequent MD/MM-PBSA stability rather than docking rank alone. Figure 3B plots consensus QSAR active probability against the best docking score for all 205 compounds and shows little global correlation between the two measures, indicating that QSAR prioritisation and docking capture complementary rather than redundant aspects of candidate quality. Compounds combining favourable docking scores with high consensus probabilities, most notably Paliperidone, Risperidone, Olaparib, and Risdiplam, therefore, emerged as the strongest candidates for downstream molecular dynamics evaluation.

At Site 2, Olaparib showed the richest interaction profile, with five hydrogen bonds across four residues (GLY67, VAL71, THR81, and VAL82), hydrophobic contacts with VAL52 and VAL82, and a halogen bond to VAL71. Paliperidone and Risperidone bind through hydrophobic contacts alone, engaging ALA76/ALA78 and VAL52/ALA69/VAL82, respectively, whereas Risdiplam makes only a single weak contact at LEU38. Site 1 interactions were generally sparser, limited to hydrophobic contacts for Olaparib and Lemborexant, with Letrozole the only ligand forming a hydrogen bond at PHE94. Overall, the strongest contacts cluster within the NAC hotspot, supporting the relevance of the Site 2 poses.

**Figure 4:**
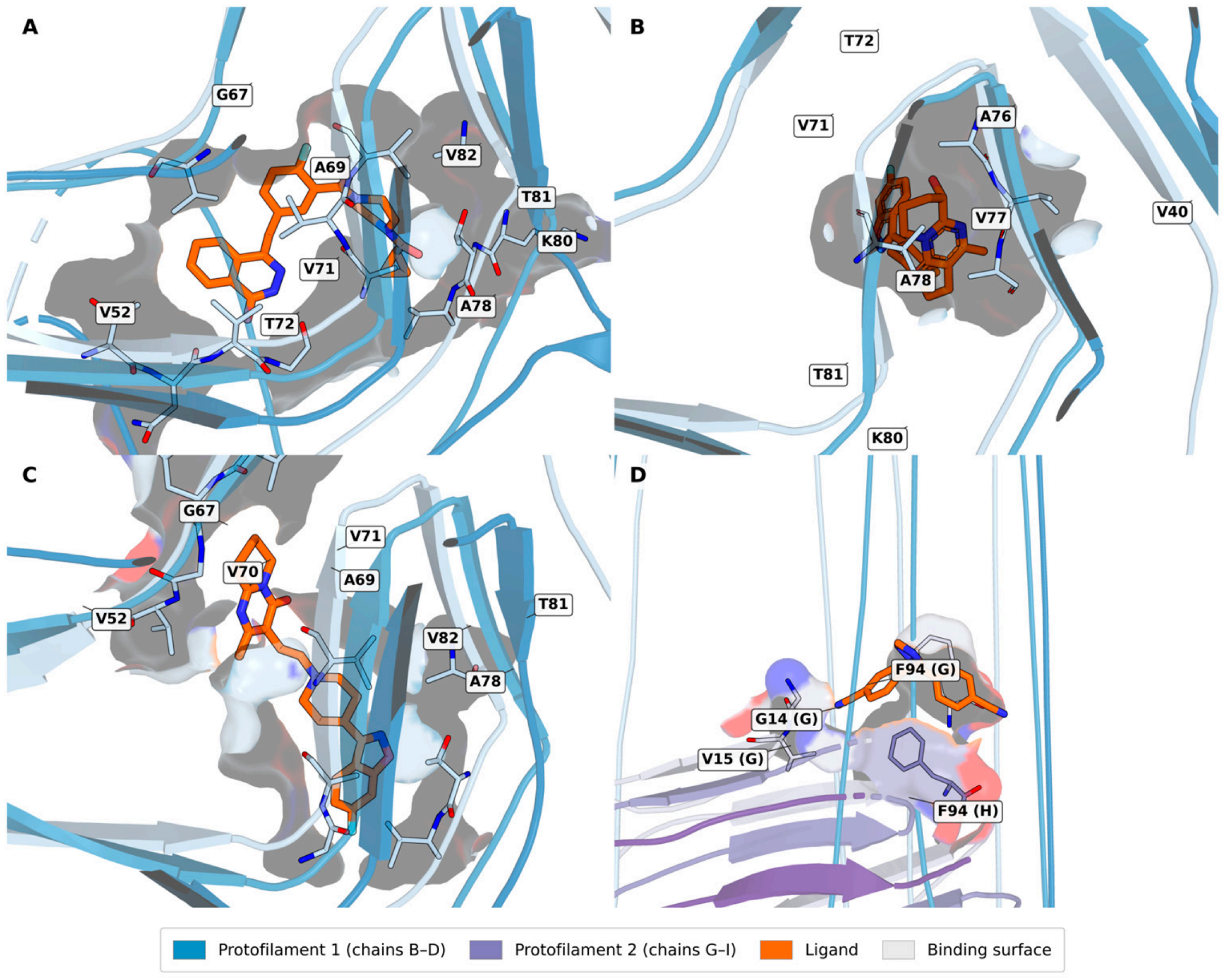
The structural binding poses of four representative compounds within the α-syn fibril (PDB 6SSX). Panels (**A**,**C**): Olaparib and Risperidone in the NAC groove (Site 2, chains B–D); the hydrogen bonds shown as red dashes. Panel (**B**): Paliperidone at the Site 2 sub-pocket. Panel (**D**): Letrozole in the Site 1 system, bound at the protofilament 2 surface. Residue contacts within 4 Å are labelled, and the protein chains are coloured by protofilament (blue: PF1; purple: PF2). Residues are numbered according to the full-length α-synuclein sequence (UniProt P37840); in (**D**) the chain identifier is given in parentheses.

Structural snapshots of four representative compounds in their simulated binding sites on the α-syn fibril are shown in Figure 4. Panels A and C illustrate Olaparib and Risperidone, respectively, nestled within the NAC groove (Site 2), formed by chains B–D, where both ligands are enveloped by a hydrophobic surface lined with the residues VAL52, ALA69, VAL71, ALA78, VAL82, and THR81. Olaparib additionally engages GLY67 and VAL71 through a network of hydrogen bonds (red dashes), consistent with its higher total contact count. Panel B shows Paliperidone occupying an adjacent sub-pocket of Site 2, primarily contacting ALA76, ALA78, and VAL77 through van der Waals interactions without forming directional hydrogen bonds. Panel D presents Letrozole in the Site 1 system, where the ligand occupies the outer surface of protofilament 2 (chains G–I, purple), contacting PHE94 of chains G and H together with GLY14 and VAL15, consistent with the PHE94 hydrogen bond listed in Table 1 and with the limited ligand retention observed for the Site 1 complexes. Concatenated multi-chain residue indices were not used for interpretation.

### 2.4. Molecular Dynamics Trajectory Analysis

#### 2.4.1. Root-Mean Square Deviation

**Figure 5:**
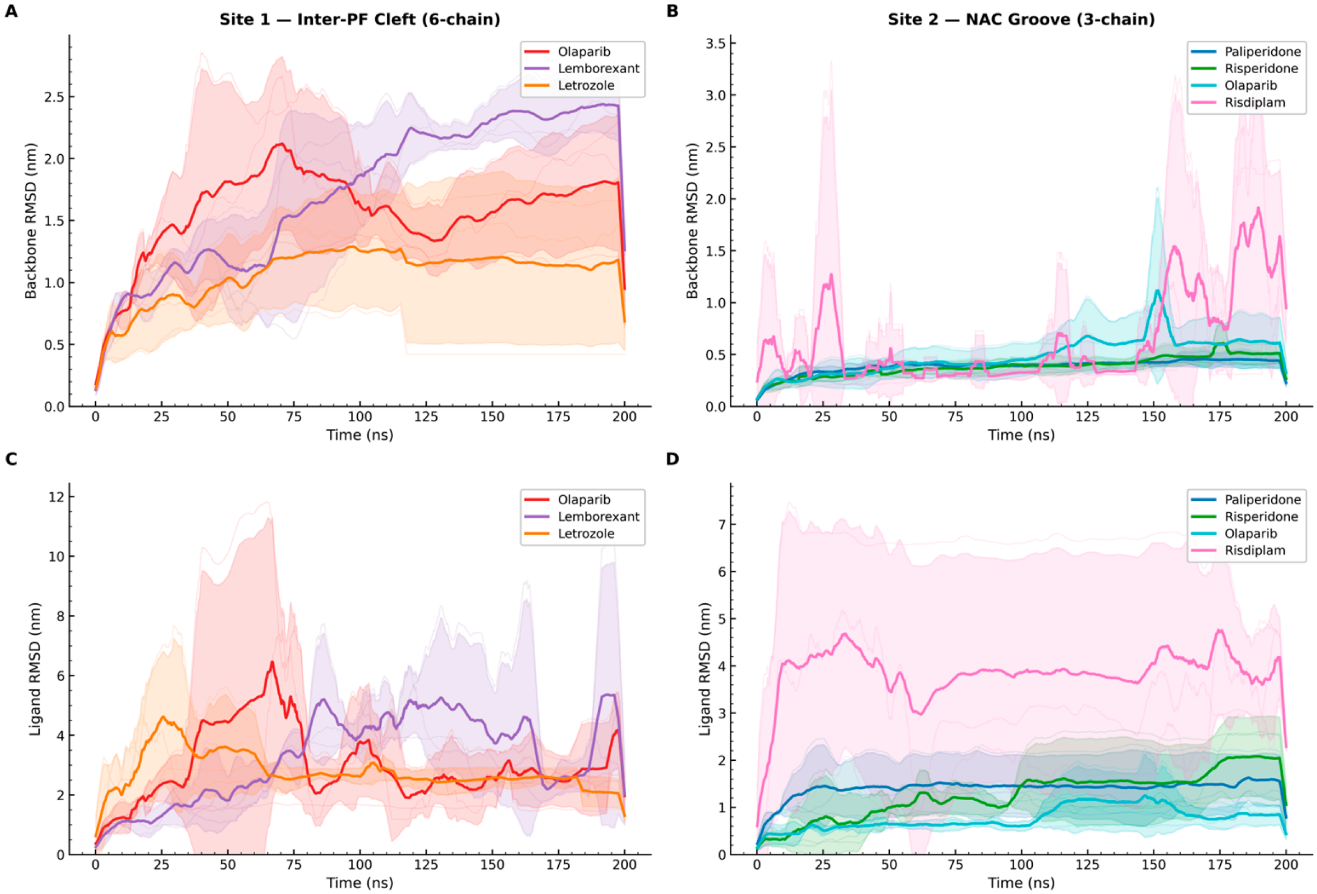
The backbone and ligand RMSD trajectories over the 200 ns MD simulations for the seven protein–ligand systems. Panel (**A**): the backbone RMSD for the Site 1 complexes (Olaparib, Lemborexant, and Letrozole). Panel (**B**): the backbone RMSD for the Site 2 complexes (Paliperidone, Risperidone, Olaparib, and Risdiplam). Panels (**C**,**D**): the ligand RMSDs at Site 1 and Site 2, respectively. The RMSDs were computed after least-squares fitting to the minimised structure.

Figure 5 shows the backbone and ligand RMSD trajectories over 200 ns as replicate means, with shaded ±SD bands across the three independent simulations. Site 2 complexes remain markedly more stable than Site 1: the backbone RMSD stays within 0.2–0.4 nm in Figure 5B, with minimal inter-replicate spread, whereas Site 1 systems in Figure 5A show larger deviations of 1.5–3.0 nm and broad variability, consistent with a more labile inter-protofilament interface. At the ligand level, Olaparib at Site 2 is the most stable, remaining below 0.5 nm throughout Figure 5D, while Paliperidone and Risperidone show only modest late drift. Risdiplam displays recurrent high-amplitude excursions and wide SD bands, consistent with repeated dissociation. In Figure 5C, all Site 1 ligands show large, poorly converged RMSDs, with Olaparib as the most striking: one replicate dissociates to ~12 nm by 75 ns and later rebinds, while the others remain displaced by 2–4 nm. Together, the tight Site 2 distributions versus the broad, replicate-dependent spread at Site 1 identify the NAC groove as the more reproducibly stable binding site.

#### 2.4.2. Root-Mean Square Fluctuation

**Figure 6:**
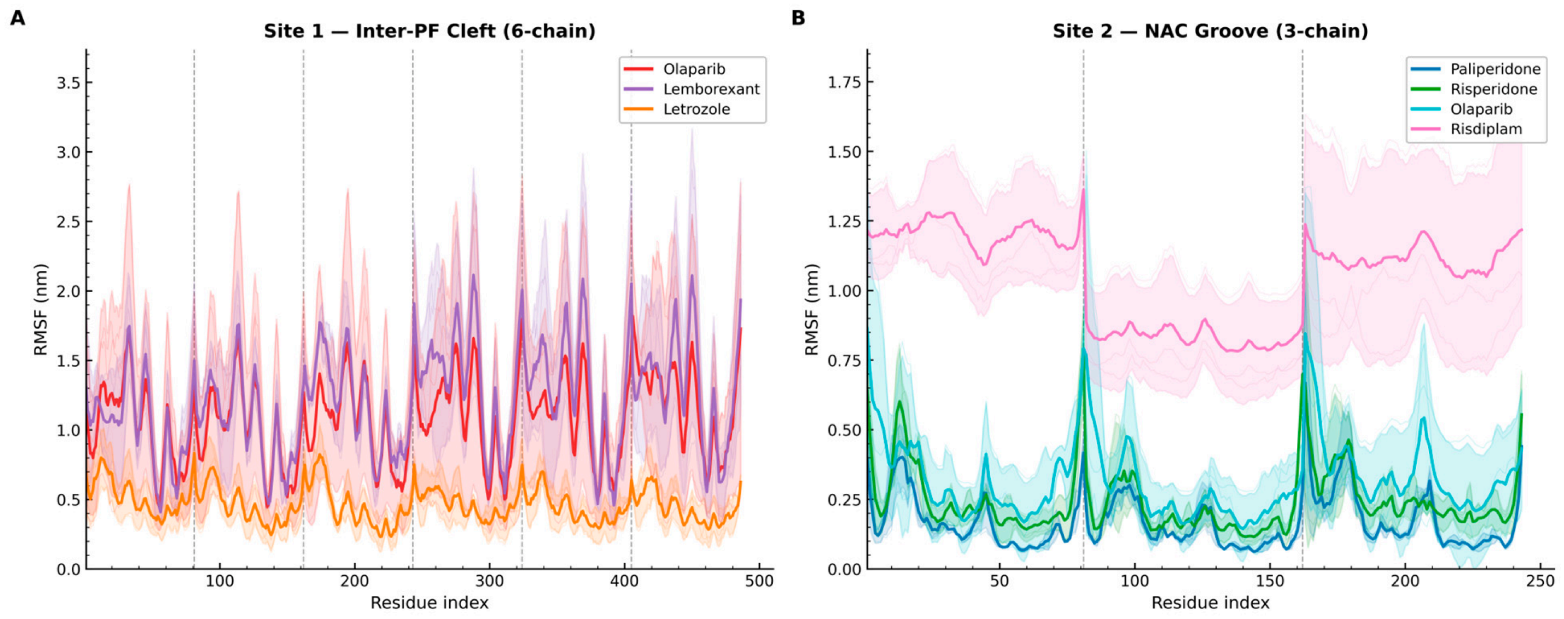
The per-residue root-mean square fluctuation (RMSF) profiles for all seven MD systems. Panel (**A**): Site 1 complexes. Panel (**B**): Site 2 complexes. The dashed vertical lines indicate the chain boundaries. The values were computed by least-squares fitting to backbone Cα atoms over the 200 ns production trajectory.

Figure 6 shows the per-residue RMSF profiles as replicate means with shaded ±SD bands across three independent simulations. In both panels, periodic peaks at the dashed chain boundaries reflect the expected mobility of terminal residues and are reproduced across replicates, indicating a structural rather than sampling-driven effect.

In Figure 6A, Letrozole shows the lowest Site 1 fluctuations, largely below 0.9 nm, Lemborexant is intermediate at roughly 0.5–1.2 nm, and Olaparib is the most mobile, with peaks of 2.5–2.7 nm and broad SD bands that reflect replicate-dependent dissociation. In Figure 6B, Paliperidone, Risperidone, and Olaparib maintain uniformly low RMSFs of 0.1–0.3 nm across the β-strand core, with only modest terminal peaks, whereas Risdiplam is the clear outlier, with elevated fluctuations of 1.0–1.5 nm across the full sequence and wide SD bands, consistent with an unstable NAC–groove complex.

#### 2.4.3. Radius of Gyration

**Figure 7:**
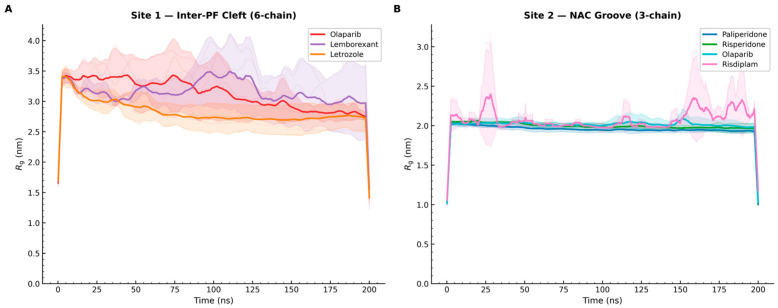
Radius of gyration (R_g_) trajectories over 200 ns MD simulations. Panel (**A**): Site 1 systems (Olaparib, Lemborexant, and Letrozole) starting at ~3.5 nm and undergoing general compaction. Panel (**B**): Site 2 systems (Paliperidone, Risperidone, and Olaparib), maintaining near-constant R_g_ of ~2.0 nm, whereas Risdiplam shows repeated excursions to 2.5–3.2 nm.

Figure 7 shows R_g_ trajectories as replicate means with shaded ±SD bands. In Figure 7B, Paliperidone, Risperidone, and Olaparib maintain a near-constant R_g_ of ~2.0 nm with minimal inter-replicate spread, indicating a stable, convergent Site 2 assembly. Risdiplam is the clear outlier, with repeated excursions to 2.5–3.2 nm and broad SD bands consistent with recurrent dissociation; occasional terminal drops are likely artefactual.

In Figure 7A, all of the Site 1 systems start near 3.5 nm and compact over time, but with much wider SD bands than Site 2, indicating genuine replicate variability. Letrozole shows the smoothest decrease to ~2.5 nm, Lemborexant follows a similar but less converged trend, and Olaparib remains the most expanded and variable, oscillating between 2.8 and 4.0 nm as dissociation-driven interface opening persists in some replicates.

#### 2.4.4. Hydrogen Bond Count

**Figure 8:**
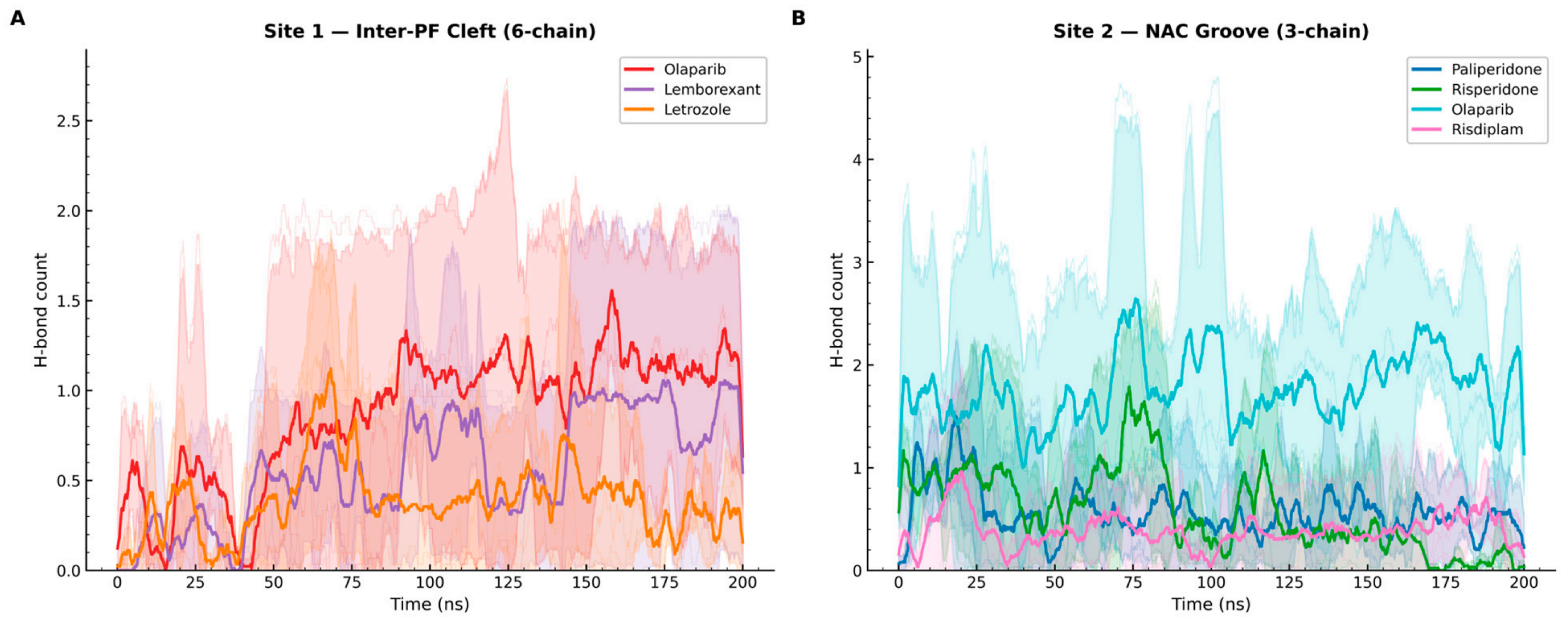
Ligand–protein hydrogen bond counts over 200 ns MD trajectories. Panel (**A**): Site 1 systems (Olaparib, Lemborexant, and Letrozole). Panel (**B**): Site 2 systems (Paliperidone, Risperidone, Olaparib, and Risdiplam). H-bond counts were computed with a donor–acceptor distance cutoff of 3.5 Å and angle cutoff of 30°.

Figure 8 shows ligand–protein hydrogen bond trajectories as replicate means with shaded ±SD bands. In Figure 8B, Olaparib sustains the strongest and most reproducible Site 2 network, maintaining ~2–5 H-bonds through most of the trajectory with relatively narrow SD bands; this trajectory-averaged range is distinct from the five hydrogen bonds observed in the representative interaction pose. Paliperidone and Risperidone form only occasional transient contacts averaging around 1, whereas Risdiplam remains near zero, with broad variability consistent with recurrent dissociation.

In Figure 8A, Site 1 H-bond counts remain low and poorly converged, with mean values rarely exceeding 1.5 and broad SD bands indicating substantial replicate variability. Lemborexant shows the most sustained signal, Letrozole forms intermittent contacts, and Olaparib displays the widest spread because one replicate remains partially bound while others dissociate. Overall, the persistent multi-H-bond network of Olaparib at Site 2 contrasts sharply with the sparse, labile contacts at Site 1 and helps explain the stronger stability of the NAC–groove complex.

#### 2.4.5. Conformational Change Analysis

**Figure 9:**
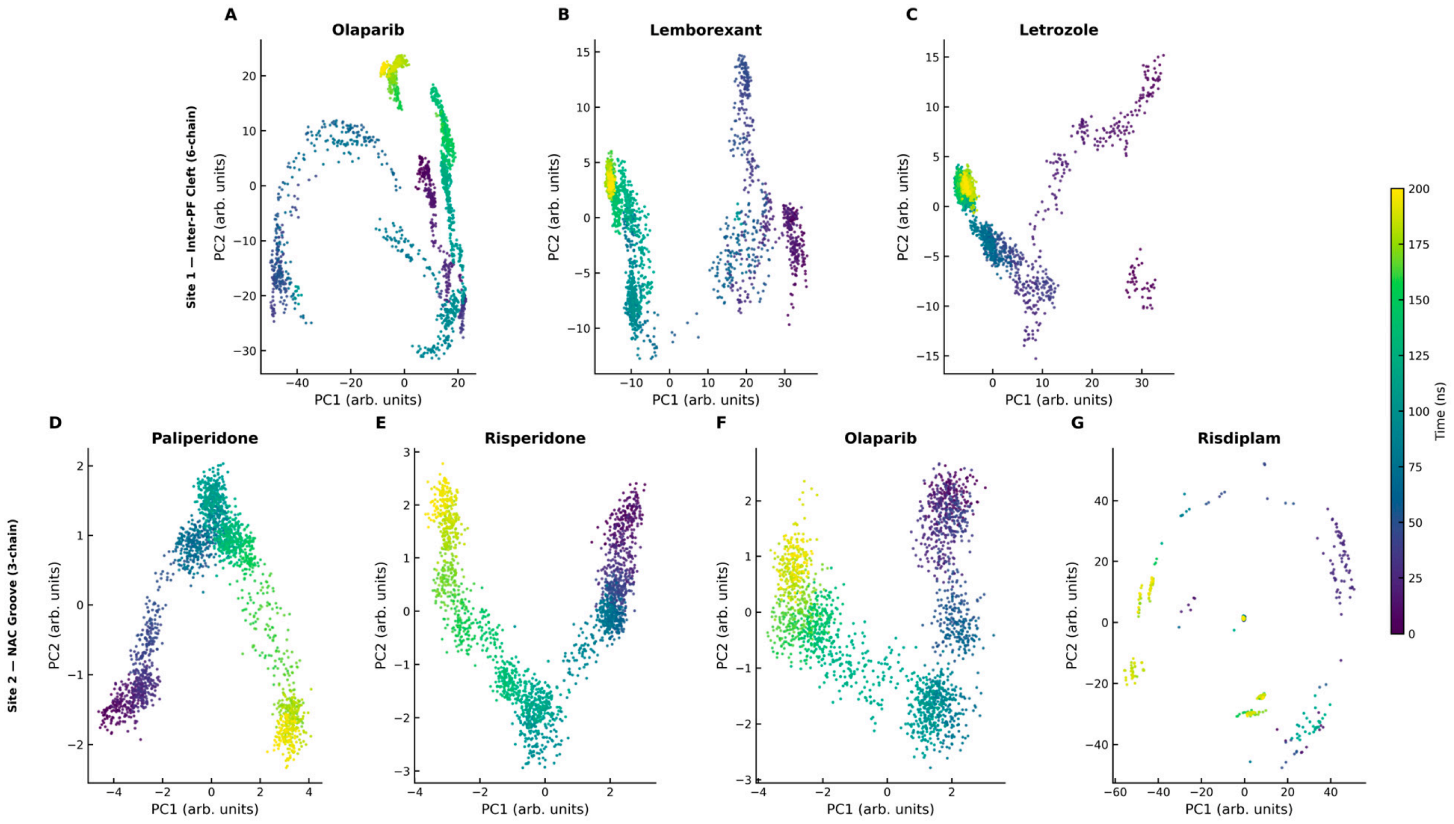
Principal component analysis (PCA) of the backbone Cα coordinates for all seven of the MD systems. Each panel shows the projection onto PC1 versus PC2, coloured by simulation time (purple = early and yellow = late). The Site 1 systems ((**A**–**C**): Olaparib, Lemborexant, and Letrozole) and Risdiplam at Site 2 (**G**) span large PC spaces (20–60 arbitrary units), while the Site 2 stable complexes ((**D**–**F**): Paliperidone, Risperidone, and Olaparib) occupy compact basins (±4 arbitrary units).

Figure 9 shows PCA projections of backbone Cα motion and reinforces the stability trends seen above. Paliperidone, Risperidone, and Olaparib at Site 2 occupy compact basins within roughly ±4 (arbitrary units), whereas Risdiplam and all of the Site 1 systems span much larger PC spaces of 20–60 (arbitrary units), consistent with greater conformational freedom and poorer convergence.

Within Figure 9, Paliperidone traces a single dense basin with smooth temporal progression, while Risperidone and Olaparib at Site 2 each show a compact two-lobed distribution consistent with a reversible transition within a still-bound state. By contrast, Risdiplam samples disconnected clusters, indicating repeated dissociation and rebinding, and Site 1 complexes remain broadly distributed, with Olaparib showing the most fragmented landscape. Together, these PCA patterns identify the NAC groove as the more stable binding environment and Olaparib at Site 2 as the most focused bound system.

#### 2.4.6. Binding Free Energy Decomposition

**Figure 10:**
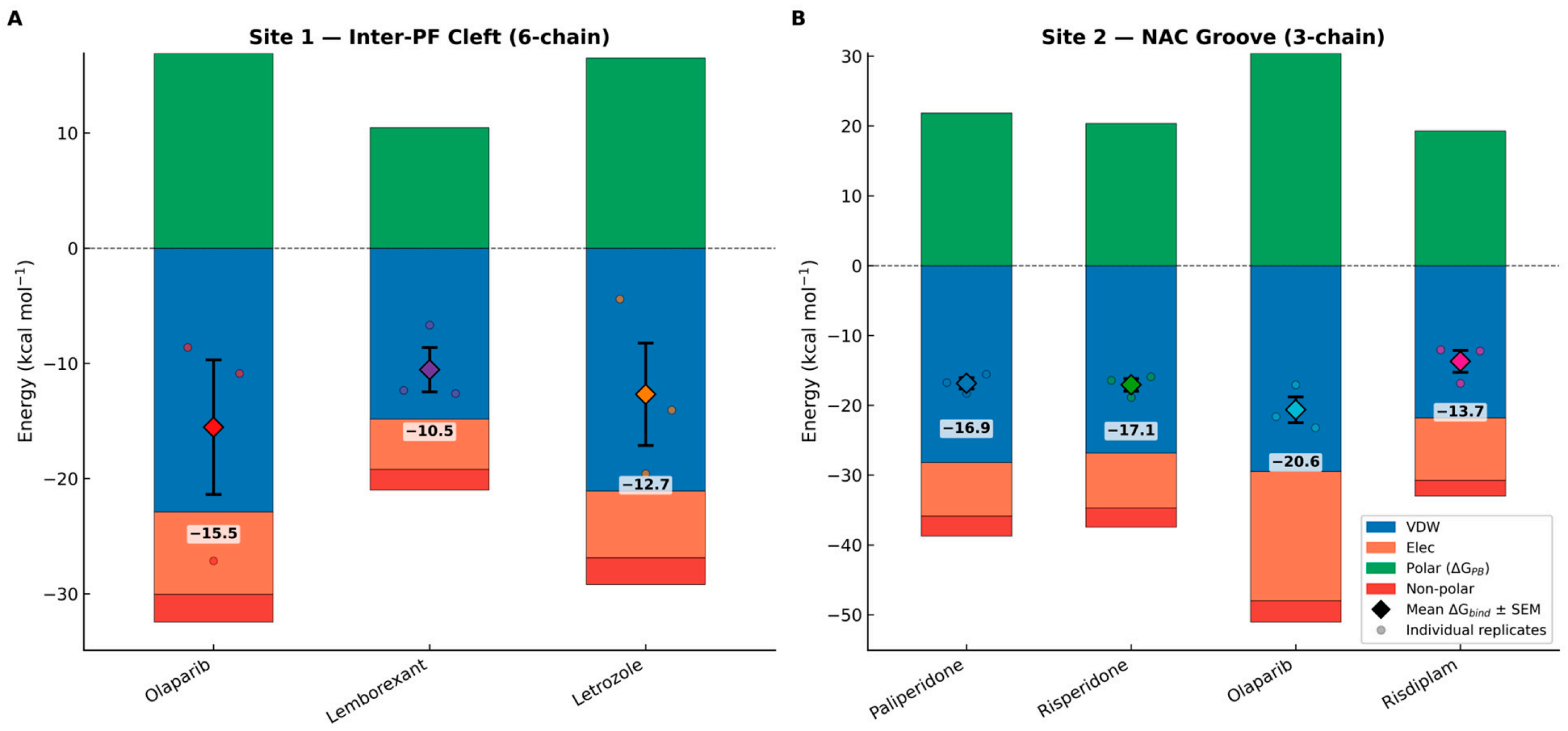
The MM-PBSA binding free energy decomposition for the seven protein–ligand systems. (**A**) Site 1, the inter-protofilament cleft (six-chain model): Olaparib, Lemborexant and Letrozole. (**B**) Site 2, the NAC groove (three-chain model): Paliperidone, Risperidone, Olaparib and Risdiplam. In both panels, the stacked bars show the per-component contributions (ΔE_vdw, ΔE_elec, ΔG_PB, and ΔG_SA); coloured diamonds with error bars give the total ΔG_bind (mean ± SEM across replicates) and small circles the individual replicate values, all taken from the final 100 ns of each 200 ns production trajectory. Colours distinguish compounds and carry no additional meaning.

Table 2 and Figure 10 summarise MM-PBSA binding free energies across the three independent 200 ns replicates per system, reported as the mean ± SEM to capture between-run reproducibility.

The Site 2 complexes show the strongest and most reproducible affinities. Olaparib is the lead compound at −20.6 ± 1.9 kcal/mol, followed by Risperidone (−17.1 ± 0.9) and Paliperidone (−16.9 ± 0.8), whereas Risdiplam is weaker at −13.7 ± 1.6. Figure 10 shows that van der Waals interactions dominate across all of the Site 2 systems, with Olaparib uniquely gaining a larger electrostatic contribution from its multi-hydrogen-bond network, while polar solvation remains the main opposing term.

The Site 1 complexes are weaker and less converged. Olaparib at Site 1 averages −15.5 ± 5.8 kcal/mol because one replicate remains bound, whereas others dissociate. Letrozole is similarly variable at −12.7 ± 4.4, and Lemborexant is the most consistent Site 1 ligand at −10.5 ± 1.9. Together, the broad Site 1 SEM values versus the tight Site 2 estimates identify the NAC groove as the more stable and druggable binding site.

## 3. Discussion

This study integrates QSAR modelling, applicability-domain gating, docking, molecular dynamics, and MM-PBSA analysis to prioritise FDA-approved drugs as candidate α-synuclein aggregation inhibitors. The main result is a consistent convergence on the NAC groove of the 6SSX fibril, with Olaparib ranking as the strongest computational lead and Paliperidone and Risperidone as reproducible secondary binders. This finding is supported by independent evidence that the PARP–α-syn axis is biologically relevant and that PARP inhibition can attenuate α-syn-driven neurotoxicity in cellular and animal models [15,16].

The QSAR results support the screening funnel. The Morgan fingerprint models performed best, with XGBoost reaching an AUROC of 0.94, consistent with the strength of gradient-boosted trees on sparse molecular fingerprints. Although the frozen ChemBERTa-77M encoder was less predictive, it captured transferable α-syn bioactivity signal without task-specific fine-tuning and greatly expanded applicability-domain coverage. The dual-AD strategy, therefore, served a central role beyond filtering, making FDA-scale repurposing tractable for a sparsely sampled target while retaining model-governed chemical-space control. Although the frozen ChemBERTa embeddings were less discriminative than the Morgan fingerprint models on this small, structurally narrow training set—reflecting the known limitations of general-purpose transformer representations applied without task-specific fine-tuning—their decisive advantage was a substantially broader yet still-calibrated applicability domain, which is precisely the property that made model-gated FDA-scale screening feasible [17,18,19].

Docking and dynamics converge on the NAC groove as the most credible binding site. Although the inter-protofilament cleft dominated the initial docking preferences, the Site 2 complexes showed stronger ligand retention, lower replicate variability, and greater stability in explicit solvent. Olaparib was the clearest example, because its docking favoured Site 1, whereas MD/MM-PBSA supported the NAC–groove Site 2 pose, maintaining its NAC–groove pose while forming the richest hydrogen-bonding network among the leading compounds. This result is biologically coherent because the NAC region nucleates α-syn aggregation, while PARP-1 activity and PAR accumulation can amplify toxic α-syn species. A PARP inhibitor that also occupies the NAC groove therefore offers a mechanistically focused hypothesis: simultaneous engagement of an aggregation-prone fibril surface and a PARP-dependent toxicity axis [15].

Experimental PARP-inhibitor studies strengthen the biological rationale for Olaparib. In the PD-relevant models, the low-toxicity PARP inhibitor analogue 10e protected neuronal cells from oxidative stress and DNA damage and reduced α-syn pre-formed fibril effects, including phosphorylated α-syn accumulation and abnormal NAD+ changes. Because 10e is a structural analogue of Olaparib with improved toxicity properties, these data support the PARP-inhibitor scaffold while cautioning that Olaparib itself may not be the safest clinical chemotype. Consistent with this axis, veliparib reduced α-syn accumulation and protected α-syn^A53T^ mice from neurodegeneration, while rucaparib-related work reported reduced α-syn accumulation in patient-derived neurons and synergy with its metabolite, M324. Together, these studies position PARP inhibition as a credible neurobiological mechanism in α-syn pathology, while leaving the optimal clinical candidate unresolved [16,20,21,22,23].

The MD and MM-PBSA results identify Site 2 as the more compelling target pocket. Olaparib was retained in the NAC groove across replicates and showed the most favourable free-energy profile, consistent with coupled hydrophobic packing and directional polar contacts within a constrained binding environment. These data extend the current PARP-inhibitor literature by proposing direct fibril engagement in addition to pathway modulation. Olaparib, therefore, represents a dual-mechanism repurposing hypothesis, combining PARP-pathway inhibition with predicted occupancy of an aggregation-relevant α-syn pocket. This mechanism remains computational and requires direct experimental validation.

Paliperidone and Risperidone provide mechanistically distinct secondary leads. Their stable Site 2 trajectories indicate that the NAC groove can accommodate chemically diverse CNS-active scaffolds, strengthening the case for druggability beyond a single PARP-inhibitor chemotype. Their dopamine D2 pharmacology is also relevant, given links between dopamine oxidation chemistry and α-syn oligomer stabilisation. However, their clinical repositioning potential is constrained by narrow neurological therapeutic windows and CNS adverse-effect liability. Critically, both Paliperidone and Risperidone are dopamine D2-receptor antagonists that are themselves clinically associated with drug-induced parkinsonism, and epidemiological and pharmacovigilance data link chronic D2-antagonist antipsychotic exposure to the worsening of parkinsonian motor signs rather than protection against Parkinson’s disease. We, therefore, do not propose them as direct antiparkinsonian therapeutics; they are presented only as structurally diverse chemical probes that illustrate the druggability of the NAC groove, and their significance should not be overstated on the basis of computational predictions alone.

A central methodological contribution is the calibrated applicability-domain strategy. The Morgan–Tanimoto domain was highly restrictive, whereas the ChemBERTa embedding domain retained most of the FDA library and enabled broader, still-gated screening. This design is particularly important for repurposing against sparsely sampled targets, where overly local similarity criteria can exclude clinically relevant chemotypes. The workflow, therefore, combines conservative molecular similarity with transferable representation learning.

Key limitations temper these conclusions. This study is computational, so all candidates require biophysical and cellular validation before therapeutic inference. Docking was performed on a single cryo-EM fibril polymorph, although α-syn fibrils are structurally heterogeneous and may vary in pocket exposure, geometry, and ligand preference. MM-PBSA supports relative ranking but does not yield exact experimental affinities, and entropic effects are approximated. Finally, the PD-oriented repositioning of Olaparib would require evidence for sufficient brain exposure and acceptable safety at neuroactive doses. More broadly, favourable docking and MM-PBSA energies indicate possible fibril engagement but do not by themselves demonstrate inhibition of α-syn aggregation, which must be established experimentally (for example, by thioflavin-T aggregation kinetics). Because only the single 6SSX polymorph was examined, future work should test whether these compounds retain stable interactions across additional α-syn fibril conformations, and the translational prioritisation of all candidates will further require assessment of blood–brain barrier permeability, clinically achievable CNS concentrations, and established safety profiles.

In summary, the NAC groove emerges as a credible α-syn target site, and Olaparib emerges as the strongest repurposing hypothesis because its predicted binding behaviour aligns with independent evidence linking PARP inhibition to α-syn toxicity. Priority validation should test Olaparib, Paliperidone, and Risperidone using ThT aggregation assays, SPR or ITC binding studies, and neuronal toxicity models, with PARP-pathway readouts included to evaluate the proposed dual mechanism.

## 4. Materials and Methods

### 4.1. QSAR Modelling

#### 4.1.1. Training Data Curation

The experimental binding affinity data for α-synuclein (UniProt P37840) were retrieved from BindingDB [24]. The activity values were converted to pKi (−log_10_ Ki, where Ki is expressed in molar units), and binary labels were assigned: active (pKi ≥ 6.0, Ki ≤ 1 μM) and inactive (pKi < 6.0). After deduplication by canonical SMILES and salt stripping using RDKit 2024.09.2 [25], the final dataset comprised 501 unique compounds (355 active and 146 inactive; 2.4:1 ratio). SMILES were standardised and validated using the MolStandardize module of RDKit 2024.09.2.

#### 4.1.2. Molecular Descriptors

Two complementary descriptor sets were computed. Morgan circular fingerprints (ECFP4-equivalent, radius = 2, 2048 bits) were generated using RDKit. In addition, all available RDKit molecular descriptors (~210, version-dependent) were calculated, including the molecular weight (MW), octanol–water partition coefficient (logP), topological polar surface area (TPSA), hydrogen-bond donor/acceptor counts (HBD/HBA), rotatable bond count, and ring system counts. The combined feature matrix was pruned by removing near-zero-variance features (variance < 0.01) and one member of each highly correlated pair (|r| > 0.95), yielding 849 final features.

#### 4.1.3. Data Splitting

To prevent information leakage across chemically similar compounds, a Murcko scaffold-based stratified split was applied. Compounds sharing the same Murcko scaffold were assigned to the same partition. The dataset was split into training (316 compounds), validation (81), and held-out test (104) sets, maintaining approximately the same active/inactive ratio across all partitions.

#### 4.1.4. QSAR Model Training and ChemBERTa-77M Encoder

Two classifiers were trained on the 849-feature Morgan descriptor matrix: Random Forest (RF; number of estimators selected from {100, 200} by 5-fold stratified cross-validation on the training set, class_weight = ‘balanced’) and Logistic Regression (C = 1.0, class_weight = ‘balanced’, solver = ‘lbfgs’), both as implemented in scikit-learn 1.8.0. Additionally, XGBoost 3.2.0 (300 estimators; scale_pos_weight = 146/355 ≈ 0.41 to correct for class imbalance) was trained and evaluated on the held-out test set. Performance was evaluated using AUROC, AUPRC, balanced accuracy, macro F1, and Matthews correlation coefficient (MCC) [26].

In parallel, ChemBERTa-77M-MLM, a RoBERTa-based transformer pre-trained on 77 million PubChem SMILES strings via masked language modelling (DeepChem/ChemBERTa-77M-MLM, https://huggingface.co/DeepChem/ChemBERTa-77M-MLM, accessed on 30 July 2026), was used as a frozen encoder without fine-tuning [27]. For each compound, SMILES strings were tokenised and processed through the 12-layer transformer, and the final-layer [CLS] token embedding (768-dimensional) was extracted as the molecular representation. Two classifiers were trained on these embeddings: RF (500 estimators, class_weight = ‘balanced’) and Logistic Regression (C = 0.1, class_weight = ‘balanced’). The ChemBERTa ensemble probability was defined as the mean of the predicted active probabilities from both classifiers.

#### 4.1.5. Applicability Domain Assessment

Reliable QSAR predictions require that query compounds reside within the chemical space of the training set (the applicability domain, AD). Two complementary AD criteria were employed. For the Morgan-based models, a compound was considered in-domain if its maximum Tanimoto similarity (ECFP4) to any training compound exceeded 0.40. The 0.40 Tanimoto cutoff reflects a widely adopted ECFP4 similarity threshold above which nearest-neighbour QSAR predictions are generally regarded as reliable, and was retained here as a conservative Morgan applicability-domain boundary. For the ChemBERTa-based models, the AD was assessed in the 768-dimensional embedding space: the mean cosine distance from each query compound to its five nearest training neighbours was computed. The AD threshold was calibrated as μ + 2σ of the within-training k = 5 nearest-neighbour cosine distance distribution, yielding a threshold of 0.367. A compound was considered in-domain if its cosine distance did not exceed this threshold. Compounds were assigned an AD tier: “strong” (within both AD definitions), “ChemBERTa-only” (within ChemBERTa AD only), or “neither” (outside both).

#### 4.1.6. Consensus Ranking

The predictions from both model families were merged on a compound identifier. The consensus probability was defined as the mean of the Morgan RF active probability and the ChemBERTa ensemble active probability. The consensus rank was computed as the mean of each compound’s rank in the respective probability-sorted lists; a lower rank indicates higher predicted activity.

### 4.2. Virtual-Screening Funnel

A three-stage sequential filter was applied to 2241 FDA-approved drugs sourced from DrugBank. Stage 1 applied the CNS permeability criteria: the molecular weight ≤ 500 Da, 1 ≤ logP ≤ 4, TPSA ≤ 90 Å^2^, and hydrogen-bond donor count ≤ 3, adapted from [28]. Stage 2 applied the QSAR filter, retaining only compounds with a consensus active probability ≥ 0.80. This ≥0.80 consensus threshold was not a universal standard value, but a deliberately stringent, study-specific cutoff chosen to enrich the docking set for high-confidence predicted actives. Stage 3 applied the ChemBERTa applicability-domain gate, retaining only compounds with cosine distance ≤ 0.367 to the training set. Compounds passing all three stages were advanced to molecular docking.

### 4.3. Structure-Based Molecular Docking

**Figure 11:**
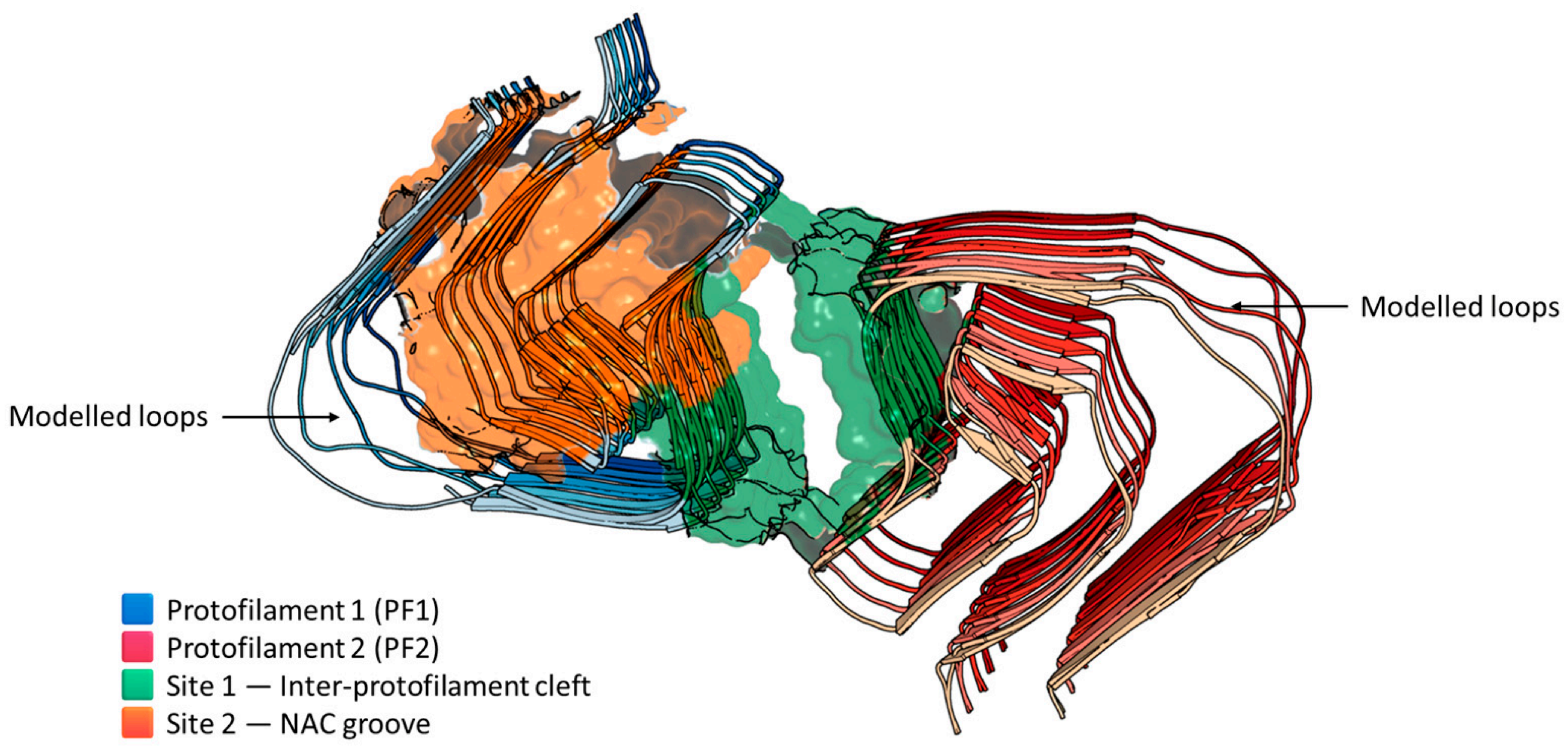
The cryo-EM structural model of the α-synuclein fibril receptor, highlighting the reconstructed loops and target binding pockets. The ribbon diagrams show Protofilament 1 (blue) and Protofilament 2 (red). The surface representations indicate the locations of the two principal binding domains: Site 1 (green, inter-protofilament cleft) and Site 2 (orange, NAC groove).

The cryo-EM α-syn fibril structure PDB 6SSX (polymorph 2A, 3.0 Å resolution, 10 chains A–J, residues 14–96) was used as the docking receptor. The missing loop regions absent from the cryo-EM density were reconstructed by computational loop modelling prior to receptor preparation, as shown in Figure 11. Two protofilaments were identified: PF1 (chains A–E) and PF2 (chains F–J). Two biologically motivated binding sites were defined: Site 1, the inter-protofilament cleft (K45–E57 interface), represented by chains B, C, D, G, H, and I, with a docking box centred at (88.45, 88.11, 86.41) Å (25 × 30 × 25 Å); and Site 2, the NAC groove (residues 61–95), represented by chains B, C, and D, with a docking box centred at (86.61, 61.39, 86.97) Å (30 × 30 × 30 Å). Disordered C-terminal residues (last two per chain) were removed prior to receptor preparation. Residues are numbered throughout according to the full-length α-synuclein sequence (UniProt P37840). Polar hydrogens and Gasteiger partial charges were added using OpenBabel 3.1.1 [29]. The PDBQT format was generated with Meeko 0.7.1 [30], with an OpenBabel fallback for any failed conversions. The Ligand 3D conformers were generated using ETKDGv3, followed by MMFF94 force field minimisation. The PDBQT format was produced with Meeko. Docking was performed with AutoDock Vina 1.2.6 [31], using exhaustiveness = 16 and num_modes = 5. An exhaustiveness of 16 was selected to provide deeper conformational sampling than the AutoDock Vina default while remaining computationally tractable across the 205-compound screen. The lowest binding energy pose was recorded for each site, and the final docking score per compound was the minimum (best) score across both sites. Molecular graphics for Figure 4 and Figure 11 were rendered with PyMOL 3.1.0 (open-source build).

### 4.4. Molecular Dynamics Simulation

Seven protein–ligand systems were prepared for all-atom molecular dynamics (MD) simulation: Paliperidone, Risperidone, Olaparib, and Risdiplam at Site 2, and Olaparib, Lemborexant, and Letrozole at Site 1. The top-ranked docking pose for each compound was used as the starting ligand conformation. Systems were selected to combine docking rank with consensus QSAR probability and to sample both binding sites, with Olaparib simulated at both sites for direct comparison.

#### 4.4.1. System Preparation

The protein–ligand complexes were parameterised using AmberTools 24.8 (University of California, San Francisco, CA, USA) [32]. The protein fibril was described with the AMBER ff14SB force field, while the ligand parameters were generated with antechamber using the GAFF2 force field and AM1-BCC partial charges, subsequently validated with parmchk2 [33]. Each complex was solvated in a cubic TIP3P water box with a minimum 12 Å buffer using tLeap, electroneutralised with Na^+^ and Cl^−^ counter-ions, then supplemented with 25 additional Na^+^ and 25 Cl^−^ ions to approximate physiological ionic strength (~150 mM NaCl).

#### 4.4.2. Simulation Protocol

All simulations were performed with OpenMM 8.3.1 [34] using a Langevin Middle integrator with a friction coefficient of 1.0 ps^−1^ under periodic boundary conditions with particle mesh Ewald electrostatics. The pressure was maintained at 1.0 bar using a Monte Carlo barostat, with a coupling frequency of 25 steps. A four-stage equilibration and production protocol was employed, with sequential harmonic positional restraints applied to backbone Cα atoms to preserve the docked conformation during equilibration, a critical measure for finite fibril fragments where exposed chain termini are susceptible to unphysical fraying. Stage 1 consisted of energy minimisation to a convergence tolerance of 10 kJ mol^−1^ nm^−1^, with backbone restraints of 10 kcal mol^−1^ Å^−2^. Stage 2 consisted of 2 ns NVT heating using a 1 fs timestep, during which the temperature was raised linearly from 5 K to 300 K over 1 ns in 30 incremental steps and then held at 300 K for 1 ns under backbone restraints of 5 kcal mol^−1^ Å^−2^. Stage 3 consisted of 10 ns NPT equilibration at a 2 fs timestep, with backbone restraints progressively released in three phases: 4 ns at 2 kcal mol^−1^ Å^−2^, 4 ns at 0.5 kcal mol^−1^ Å^−2^, and 2 ns fully unrestrained. Stage 4 consisted of 200 ns NPT production at a 2 fs timestep, with the protein backbone fully free; the terminal chain Cα atoms were restrained at 1 kcal mol^−1^ Å^−2^ to prevent end-fraying of the finite fibril segment. For the 3-chain Site 2 systems, chains B and D were restrained; for the 6-chain Site 1 systems, chains B, D, G, and I were restrained. Coordinates were saved every 100 ps, and energetic properties every 10 ps. To assess convergence and binding reproducibility, two additional independent replicates were performed for each of the seven systems using different random velocity seeds, yielding three 200 ns trajectories per compound. All MD-derived metrics were reported as the mean across replicates, and binding free energies were reported as the mean ± SEM.

#### 4.4.3. Trajectory Analysis

Production trajectories were converted to the GROMACS format and analysed using GROMACS 2025.4 [35] tools. The backbone RMSDs and per-residue RMSFs were computed after least-squares fitting to the minimised structure. The radius of gyration, solvent-accessible surface area, protein–ligand hydrogen bonds, and the principal component analysis of the backbone Cα coordinates were calculated using the corresponding GROMACS modules. Binding free energies were estimated by MM-PBSA using gmx_MMPBSA v1.6.5 [36], applied to the final 100 ns of each production trajectory, decomposing the ΔG_bind into van der Waals, electrostatic, polar solvation (Poisson–Boltzmann), and non-polar solvation contributions.

#### 4.4.4. Binding Free Energy Calculations

Binding free energies were estimated using the MM-PBSA method, as implemented in gmx_MMPBSA v1.6.5 [36], applied directly to the GROMACS trajectories using the single-trajectory approach. A total of 200 evenly spaced snapshots were extracted from the final 100 ns (stabilised window) of each 200 ns production trajectory for energy evaluation, consistent with the MM-PBSA sampling window described above. The total binding free energy (ΔG_bind) and its van der Waals, electrostatic, polar solvation, and non-polar solvation components were reported as mean ± SEM across replicates. Per-residue energy decomposition was performed to identify the key binding-site residues contributing to ligand affinity.

## 5. Conclusions

This study establishes an integrated computational framework for repurposing FDA-approved drugs as candidate α-syn aggregation inhibitors, combining QSAR modelling, calibrated applicability-domain gating, docking, replicated molecular dynamics, and MM-PBSA analysis. ChemBERTa embedding substantially expanded reliable FDA-library coverage relative to Morgan–Tanimoto similarity, from 5.5% to 76.8%, enabling broad but model-gated screening of approved compounds. This workflow prioritised 205 docked candidates and advanced seven systems to triplicate the 200 ns simulations for dynamic stability assessment.

Across docking, MD, and MM-PBSA, the NAC groove emerged as the dominant druggable site on the α-syn fibril surface. Olaparib was the strongest lead, despite a stronger raw docking score at Site 1, with the most favourable replicate-averaged binding free energy (−20.6 ± 1.9 kcal/mol) and the richest contact network, including five hydrogen bonds, a halogen bond, and hydrophobic interactions. Risperidone (−17.1 ± 0.9 kcal/mol) and Paliperidone (−16.9 ± 0.8 kcal/mol) formed reproducible secondary Site 2 complexes with narrow inter-replicate uncertainty. In contrast, the Site 1 complexes showed poorer ligand retention and greater replicate variability. These results nominate the NAC groove as the primary target pocket and identify Olaparib, Risperidone, and Paliperidone as clinically approved, CNS-relevant candidates for experimental testing.

Priority validation should test these candidates using thioflavin-T aggregation assays, solution-state NMR epitope mapping, surface plasmon resonance binding studies, and neuronal toxicity models. Future work should expand α-syn bioactivity datasets, fine-tune ChemBERTa for task-specific prediction, and apply enhanced sampling or free energy perturbation to refine affinity estimates and guide lead optimisation.

## Figures and Tables

**Table 1:** The molecular interaction profiles of the top-ranked drug candidates at the α-synuclein fibril binding sites. The table details specific residue engagements, hydrogen bond donor–acceptor distances (*d*(H···A)), hydrophobic contacts, and auxiliary bonds across the MD-equilibrated systems for both the inter-protofilament cleft (Site 1) and the NAC groove (Site 2). Total contacts (*n*) denotes all ligand–protein contacts within 4 Å, including those not listed individually.

Compound	Site	H-Bonds	H-Bond d(H···A) (Å)	Hydrophobic Contacts	Halogen/Other	Total Contacts (*n*)
Olaparib	Site 1	—	—	VAL16 (3.72), ALA53 (3.58)	—	4
Lemborexant	Site 1	—	—	VAL48 (3.80), VAL55 (3.30)	—	2
Letrozole	Site 1	PHE94	3.00	PHE94 (3.58)	—	7
Paliperidone	Site 2	—	—	ALA76 (3.95), ALA78 (3.80)	—	2
Risperidone	Site 2	—	—	VAL52 (3.93), ALA69 (3.54), VAL82 (3.63)	—	4
Olaparib	Site 2	GLY67 (×2), VAL71, THR81, VAL82	1.92, 1.88, 1.98, 3.65, 2.14	VAL52 (3.67), VAL82 (3.89)	Halogen bond: VAL71 d = 3.43	9
Risdiplam	Site 2	—	—	LEU38 (3.88)	—	1

**Table 2:** The replicate-averaged MM-PBSA binding free energies (ΔG_bind_) for the seven protein–ligand systems, reported as the mean ± SEM across the three independent 200 ns replicates.

Compound	Site	ΔG_bind (kcal/mol, Mean ± SEM)
Olaparib	Site 2	−20.6 ± 1.9
Risperidone	Site 2	−17.1 ± 0.9
Paliperidone	Site 2	−16.9 ± 0.8
Risdiplam	Site 2	−13.7 ± 1.6
Olaparib	Site 1	−15.5 ± 5.8
Letrozole	Site 1	−12.7 ± 4.4
Lemborexant	Site 1	−10.5 ± 1.9

## Data Availability

The datasets, screening outputs, molecular docking results, molecular dynamics analysis files, and associated Supplementary Materials supporting the findings of this study are available through Zenodo at: https://zenodo.org/records/20679035 (accessed on 30 July 2026).

## Supplementary Materials

The following supporting information can be downloaded at: https://www.mdpi.com/article/10.3390/ijms27157025/s1.
