# Supplementary material for: QSAR-Guided Virtual Screening and Molecular Dynamics Reveal Olaparib as a Repurposing Lead Against α-Synuclein Aggregation
Source: Int J Mol Sci. 2026 Aug 5;27(15):7025. doi: 10.3390/ijms27157025 (PMC13467068; doi:10.3390/ijms27157025)
Supplement: Supplementary file 1 [file ijms-27-07025-s001.zip › ijms-4425670-supplementary.pdf]

Table S1. Performance metrics of the Morgan- and ChemBERTa-based QSAR models on the held-out scaffold-stratified test set (n = 104). Reported metrics include AUROC, AUPRC, balanced accuracy, and F1 score.

Model	AUROC	AUPRC	Balanced Acc.	F1 score
RF (Morgan)	0.93	0.96	0.64	0.82
LR (Morgan)	0.88	0.93	0.80	0.87
**Morgan Ensemble**	**0.94**	**0.97**	**0.81**	**0.87**
RF (ChemBERTa)	0.77	0.88	0.64	0.89
LR (ChemBERTa)	0.77	0.88	0.71	0.87
**ChemBERTa Ensemble**	**0.79**	**0.89**	**0.73**	**0.88**

Table S2. Top-20 FDA-approved drug candidates ranked by their best AutoDock Vina docking score against the α-synuclein fibril structure PDB 6SSX. For each compound, the table reports the best score overall, site-specific scores for Site 1 and Site 2, consensus QSAR probability, molecular weight, and logP.

Rank	Compound	Best Score (kcal/mol)	Score Site 1	Score Site 2	Consensus Prob.	MW (Da)	logP
1	Olaparib	−7.91	−7.91	−6.96	0.809	434.5	1.96
2	Paliperidone	−7.75	−7.75	−6.73	0.832	426.5	1.76
3	Niraparib	−7.18	−7.18	−6.29	0.861	320.4	2.47
4	Dordaviprone	−7.06	−7.06	−5.97	0.807	386.5	3.05
5	Parecoxib	−6.89	−6.89	−6.06	0.821	370.4	3.51
6	Risperidone	−6.88	−6.57	−6.88	0.834	410.5	2.63
7	Flibanserin	−6.73	−6.73	−6.10	0.811	390.4	3.83
8	Pemirolast	−6.72	−6.72	−5.12	0.886	228.2	1.46
9	Risdiplam	−6.71	−6.71	−6.67	0.810	401.5	1.18
10	Olutasidenib	−6.65	−6.65	−6.03	0.888	354.8	1.89
11	Mizolastine	−6.57	−6.47	−6.57	0.889	432.5	3.73
12	Laropiprant	−6.52	−6.52	−5.77	0.846	435.9	3.87
13	Crizotinib	−6.50	−6.50	−5.98	0.828	450.3	3.57
14	Mitapivat	−6.49	−6.49	−5.93	0.850	450.6	1.62
15	Leniolisib	−6.37	−6.37	−6.13	0.829	450.5	2.01
16	Duvelisib	−6.36	−6.36	−6.05	0.847	416.9	3.67
17	Triazolam	−6.34	−6.34	−5.28	0.882	343.2	3.63
18	Lemborexant	−6.32	−6.32	−6.10	0.836	410.4	3.34
19	Brotizolam	−6.28	−6.28	−5.34	0.868	393.7	3.90
20	Carprofen	−6.26	−6.26	−5.60	0.837	273.7	3.88
